# Adult IDH Wild-Type Glioblastoma Ultrastructural Investigation Suggests a Possible Correlation between Morphological Biomarkers and Ki-67 Index

**DOI:** 10.3390/biomedicines11071968

**Published:** 2023-07-12

**Authors:** Pietro Familiari, Michela Relucenti, Pierfrancesco Lapolla, Mauro Palmieri, Manila Antonelli, Loredana Cristiano, Claudio Barbaranelli, Myriam Catalano, Luca D’Angelo, Giuseppe Familiari, Antonio Santoro, Alessandro Frati, Placido Bruzzaniti

**Affiliations:** 1 Department of Human Neurosciences, Division of Neurosurgery, Policlinico Umberto I University Hospital, Sapienza University of Rome, 00185 Rome, Italy; pietro.familiari@uniroma1.it (P.F.); pierfrancesco.lapolla@nds.ox.ac.uk (P.L.); mauro.palmieri@uniroma1.it (M.P.); lucadangelo_80@hotmail.com (L.D.); antonio.santoro@uniroma1.it (A.S.); alessandro.frati@uniroma1.it (A.F.); placido.bruzzaniti@uniroma1.it (P.B.); 2 Department of Anatomy, Histology, Forensic Medicine, and Orthopedics, Sapienza University of Rome, 00185 Rome, Italy; giuseppe.familiari@uniroma1.it; 3 Nuffield Department of Surgical Sciences, University of Oxford, Oxford OX3 9DU, UK; 4 Department of Radiological, Oncological and Anatomo-Pathological Sciences, Sapienza University of Rome, 00185 Rome, Italy; manila.antonelli@uniroma1.it; 5 Department of Life, Health and Environmental Sciences, University of L’Aquila, 67100 L’Aquila, Italy; loredana.cristiano@univaq.it; 6 Department of Psychology, Sapienza University of Rome, 00185 Rome, Italy; claudio.barbaranelli@uniroma1.it; 7 Department of Physiology and Pharmacology “Vittorio Erspamer”, Sapienza University of Rome, 00185 Rome, Italy; myriam.catalano@uniroma1.it; 8 Department of Neurosurgery, Istituto di Ricovero e Cura a Carattere Scientifico Neuromed, 86077 Pozzilli, Italy; 9 Fabrizio Spaziani Hospital, 03100 Frosinone, Italy

**Keywords:** microglia, glioblastoma, EVs, biomarker, Ki67, lipid vesicles

## Abstract

Glioblastoma is an aggressive brain tumor with an average life expectancy between 14 and 16 months after diagnosis. The Ki-67 labeling index (LI), a measure of cellular proliferation, is emerging as a prognostic marker in GBM. In this study, we investigated the ultrastructure of glioblastoma tissue from 9 patients with the same molecular profile (adult IDH wild-type glioblastoma, wild-type ATRX, and positive for TP53 expression, GFAP expression, and EGFR overexpression) to find possible ultrastructural features to be used as biomarkers and correlated with the only parameter that differs among our samples, the Ki-67 LI. Our main results were the visualization of the anatomical basis of astrocyte-endothelial cells crosstalk; the ultrastructural in situ imaging of clusters of hyperactivated microglia cells (MsEVs); the ultrastructural in situ imaging of microglia cells storing lipid vesicles (MsLVs); the ultrastructural in situ imaging of neoplastic cells mitophagy (NCsM). The statistical analysis of our data indicated that MsEVs and MsLVs correlate with the Ki-67 LI value. We can thus assume they are good candidates to be considered morphological biomarkers correlating to Ki-67 LI. The role of NCsM instead must be further evaluated. Our study findings demonstrate that by combining ultrastructural characteristics with molecular information, we can discover biomarkers that have the potential to enhance diagnostic precision, aid in treatment decision-making, identify targets for therapy, and enable personalized treatment plans tailored to each patient. However, further research with larger sample sizes is needed to validate these findings and fully utilize the potential of ultrastructural analysis in managing glioblastoma.

## 1. Introduction

Glioblastoma is a type of brain cancer that is highly malignant; it represents up to 48.6% of the primary malignant tumor in the central nervous system. Despite the advances in neurosurgery, radiation therapy, and chemotherapy, the median survival time of glioblastoma patients still ranges from 9 to 16 months [1]. Although individual differences exist, with some patients surviving only for a few months and others for years [2]. Wild-type isocitrate dehydrogenase (IDH) glioblastoma is mostly a primary or de novo tumor that occurs in people over the age of 50 who have a brief clinical history (less than three months before diagnosis) and no previously present lower-grade glioma. [3]. IDH-wildtype refers to an associated genetic profile characteristically altered. [4]. Genetic/molecular markers related to Glioblastoma are ATRX mutation, TP53 expression, GFAP, and EGFR.

ATRX [5] is a chromatin remodeler protein, recurrently mutated in WHO grade II/III astrocytic glioma and secondary glioblastoma [6,7,8], where 75% of astrocytic gliomas with IDH1 and TP53 mutations also carry ATRX mutations [6]).

TP53 is one of the most commonly deregulated genes in cancer. The p53-ARF-MDM2 pathway is deregulated in 84% of glioblastoma patients and 94% of glioblastoma cell lines. Deregulated p53 pathway components have been implicated in glioblastoma cell invasion, migration, proliferation, evasion of apoptosis, and cancer cell stemness [9].

Glial fibrillary acidic protein (GFAP) is an intermediate filament protein found in gliomas that astrocytes and neural progenitor cells generate. Changes in GFAP alternative splicing are linked to glioma malignancy. The canonical isoform GFAP is downregulated in higher-grade cancers, resulting in greater GFAP isoform dominance in the network [10].

Glioblastoma frequently has epidermal growth factor receptor (EGFR) mutations. EGFR activation can be increased in a variety of ligand-dependent and ligand-independent ways. Evidence suggests that EGFR is overexpressed in most primary glioblastomas and some of the secondary glioblastomas and is characteristic of more aggressive glioblastoma phenotypes. Mutations of EGFR occur in roughly one-third of all classical tumors and often in mesenchymal, proneural, and neural glioblastomas [11].

Ki-67 is a nuclear protein; it reflects the physiological proliferative state of the cells in which this marker is expressed [12]. Different studies have investigated the prognostic potential of the Ki-67 labeling index (LI) in gliomas [13]. The Ki-67 increases with increasing WHO grade [14,15], it was demonstrated that there was no difference in median Ki-67 LI between IDH-mutated and IDH-wildtype glioblastomas (*p* = 0.9) and Ki-67 LI was not associated with survival in glioblastomas in either univariate (*p* = 0.9) or multivariate analysis including MGMT promoter methylation status and excluding IDH-mutated glioblastomas (*p* = 0.2). Ki-67 LI may be of value in the differential diagnostic setting, but it must not be over-interpreted in the clinicopathological context. In the innovative paper [16], the authors provide an initial study on the predictive value using multiple MRI characteristics for Ki-67 LI in glioma. Their results demonstrate that the degree of peritumoral edema was more severe in the group with high Ki-67 LI, which also suggested that there is a certain correlation between peritumoral edema and Ki-67 LI, both of which are related to the higher malignant degree of glioma.

Recent studies focus on the glioblastoma microenvironment, which consists of an extracellular matrix, interstitial fluid, and non-neoplastic cells compartmentalized in anatomically-distinct regions called “tumor niches”, in which glioma stem cells are localized [17,18,19]. Multiple elements influence intercellular communication in this intricate microenvironment. Extracellular vesicles (EVs), small membrane-bound particles produced by cells, have emerged as an ultrastructural morphological biomarker in brain malignancies in recent years [20,21,22,23,24,25].

Mitophagy is an additional ultrastructural morphological biomarker that could relate to biomolecular data, the process by which dysfunctional or damaged mitochondria are selectively removed from the cell. Mitophagy is an essential mechanism for maintaining cellular homeostasis that prevents the accumulation of damaged mitochondria, which increases cell death or senescence [26]. The role of mitophagy in glioblastoma appears to be complex and context-dependent. Some studies indicate that inhibition of mitophagy in glioblastoma cells results in decreased proliferation and increased sensitivity to radiation therapy, and another study found that activation of mitophagy in glioblastoma cells resulted in decreased tumor growth and increased survival in a mouse model [27]. More research is needed to fully understand the mechanisms underlying the effects of mitophagy in glioblastoma.

The presence of cells containing a discrete amount of lipid vesicles is another ultrastructural feature that may be related to biomolecular markers. Lipid vesicles are considered dynamic organelles that store and mobilize lipids in cells. It has been proven that glioblastoma cells with high amounts of lipid droplets are more resistant to chemotherapy and radiation therapy than cells with low lipid droplets. In addition, it was observed that in glioblastoma cells, the inhibition of lipid droplet formation can sensitize these cells to chemotherapy [28]; thus, their presence promotes therapy resistance. On the other hand, lipid droplets have also been implicated in promoting tumor growth and invasion in glioblastoma, being possible that lipid droplets can be transferred from glioblastoma cells to endothelial cells, promoting angiogenesis and tumor growth [29,30].

Ultrastructural studies on glioblastoma architecture exist, but no one focuses on the possible relation between ultrastructural characteristics and molecular biomarkers. We investigated the ultrastructure of glioblastoma tissue from 9 patients with the same molecular profile (adult IDH wild-type glioblastoma, wild-type ATRX, and positive for TP53 expression, GFAP expression, and EGFR overexpression) to find possible ultrastructural features to be used as biomarkers and correlated with the only parameter that differs among our samples, the Ki-67 labeling index.

## 2. Materials and Methods

### 2.1. Patients Characteristics

Four males (age 58 ± 7.4) and five females (age 54.8 ± 6.9) were diagnosed with Glioblastoma Grade IV CNS-WHO2021, IDH wild type. A neuropathologist reviewed hematoxylin and eosin-stained formalin-fixed paraffin-embedded sections for confirmation of a diagnosis of glioblastoma WHO grade IV-wild type as per 2021 World Health Organization criteria (Supplementary Materials Table S1).

### 2.2. Histopathological Procedures and Molecular Characterization of Glioblastoma Tumors

Hematoxylin and eosin staining: FFPE 3-mm-thick sections from tissue samples were cut, deparaffination with xylol, and stained with hematoxylin and eosin.

Immunohistochemistry: an immunohistochemical panel in which GFAP, ATRX, P53, IDH1 R132H, and Ki67 were utilized. This panel is routinely performed in Policlinico Umberto 1 Hospital according to the key diagnostic genes, molecules, pathways, and/or combinations in the 2021 WHO Classification of CNS Tumors [3,4].

The procedure was performed on Leica BOND-III fully automated IHC stainer (Leica Biosystems, Nussloch, Germany). The protocol was executed: tissue sections were cut at 5 μm, dried at 70 °C for 30 min, and then dewaxed. The Bond Rx system with Epitope Retrieval Solution 1 (pH 6) for 30 min was used for antigen retrieval. Sections were incubated for 30 min with GFAP (Leica Novocastra, Milan Italy, clone GA5, mouse monoclonal, 1:400 dilution), ATRX (NBP1-83077, rabbit polyclonal, 1:1000 dilution; Novus Biologicals, Milan, Italy), p53 (NCL-L-p53-DO7, mouse polyclonal, 0.875 μg/mL; Leica Novocastra, Milan, Italy), IDH1 R132H (DIA H09, mouse monoclonal antibody, Dianova, BIOZOL, Hamburg, Germany), Ki64 (NCL-L-Ki67-MM1—mouse monoclonal, 84 mg/L, 1:200 dilution, Leica Novocastra, Milan, Italy), Ki67 Clone MIB-1 and anti-EGFR Antibody (clone RM240, 1:150; RevMAb Bioscences, Burlingame, CA, USA).

### 2.3. Light and Transmission Electron Microscopy Protocols

Samples were fixed in 2.5% glutaraldehyde in PB 0.1 M pH 7.4 for at least 48 h at 4 °C and then rinsed with PB [31]. Samples were then post-fixed with OsO_4_ 1.33% (Agar Scientific, Stansted, UK) for 2 h, washed with PB for 20 min, and incubated with 1% tannic acid (Sigma-Aldrich, St. Louis, MO, USA) in dH_2_O for 30 min. Dehydration in ascending ethanol series (30%, 70%, 95%, 100% *v*/*v* × 3) was performed. Ethanol substitution with propylene oxide was performed (BDH Italia, Milan, Italy), and then samples were embedded in a mixture of 50:50 propylene oxide and epoxy resin Agar 100 (SIC, Rome, Italy) overnight at 25 °C under a chemical fume hood. Finally, samples were embedded in Agar 100 resin and put in a stove at 60 °C for 48 h. Semithin sections (1 µm thick) were collected on glass slides, stained blue by Azur II, to perform light microscopy observations in a light microscope (Carl Zeiss Axioskop-40, Zeiss, Oberkochen, Germany). Light microscopy observation of 1 µm thick epoxy resin semithin sections allows imaging at high magnification [32]. Then, ultrathin sections (80–90 nm), for transmission electron microscopy (TEM) observation, were cut using an ultramicrotome (Leica EM UC6, Vienna, Austria). Ultrathin sections were collected on 100-mesh copper grids (Assing, Rome, Italy) stained with Uranyless© solution (Electron Microscopy Sciences, Hatfield, PA, USA) and led citrate [33,34]. Imaging was performed using a transmission electron microscope set with an accelerating voltage of 60 kV (Carl Zeiss EM10, Thornwood, NY, USA). Images were acquired with a CCD digital camera (AMT CCD, Deben UK Ltd., Suffolk, UK) [35].

### 2.4. Semiquantitative Evaluation of Ultrastructural Characteristics

The semiquantitative evaluation of ultrastructural morphological characteristics was performed by examining pictures from 30 microscopic fields captured at 2000× for each sample. Microglia cells showing EVS secretion (MsEVs), Microglia cells storing lipid vesicles (MsLVs), and neoplastic cells mitophagy (NCsM) were evaluated as absent or present in each image. The overall percentage of positivity was reported for each parameter and each patient in Table S4 of Supplementary Materials. Statistical analysis of data was performed by MedCalc© software (version 20.218, MedCalc Software ltd, Ostend, Belgium) and SPSS© statistical software (version 29, IBM©-SPSS© Statistics, Milan, Italy) [36,37].

## 3. Results

### 3.1. Histopathological and Immunohistochemical Analysis

**Figure 1:**
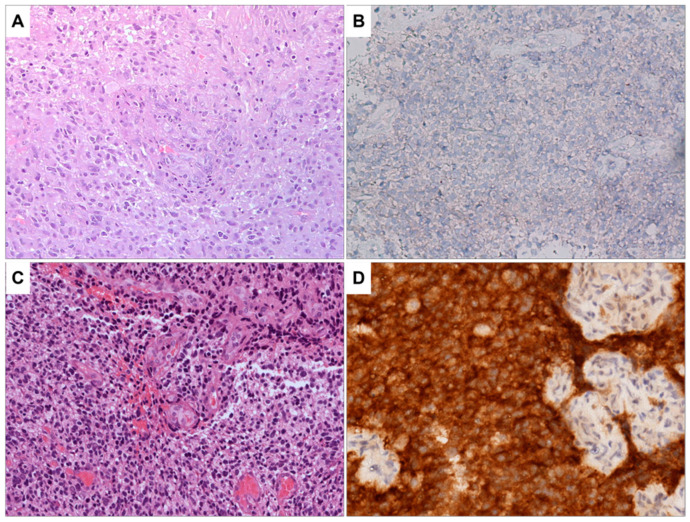
(**A**) Glioblastoma, *IDH* wild type. H&E staining, the tumor is highly cellular; microvascular proliferation is evident in the center of the image (100×). (**B**) Immunohistochemistry for IDH^R232H^ is negative in neoplastic cells (200×). No orange staining is visible. (**C**) As a control sample, a glioblastoma, IDH mutant is shown. H&E staining, this sample shows pleomorphic and hyperchromatic neoplastic cells. Microvascular proliferation is present (100×). (**D**) As a control sample for positivity for IDH^R232H^, a glioblastoma IDH mutant is presented. This sample is heavily stained in orange, indicating that IDH^R232H^ is positive in neoplastic cells (200×).

**Figure 2:**
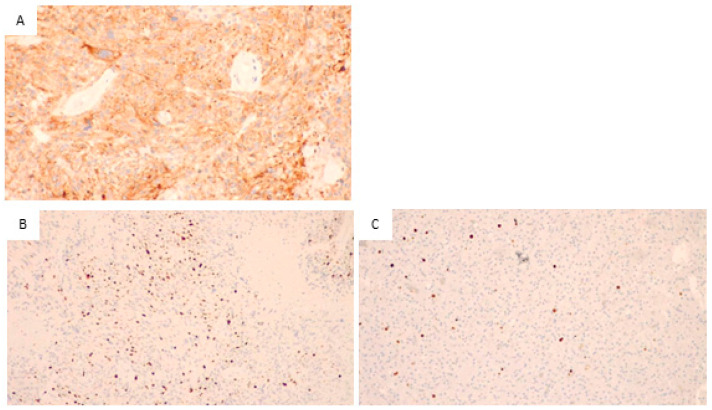
Glioblastoma, IDH wild type. (**A**) Immunohistochemical staining for EGFR, the neoplastic cells positive for EGFR are stained in orange (200×), so the sample is positive for EGFR. (**B**) Immunohistochemical staining for Ki67, numerous cells positive for Ki67 (stained in orange) are visible. Thus, a high proliferative activity is present (200×). (**C**) In the control sample, immunohistochemical staining for Ki67, in this sample is evident the presence of a few proliferative cells with orange staining. This indicates a low proliferation index.

The results of the molecular characterization of glioblastoma samples are summarized in Table S2 of the Supplementary Materials section. All the patients were negative for ATRX mutation and positive for TP53 and GFAP expression and showed EGFR hyperexpression. The value of Ki-67 ranges from 10% to 40%. The histopathological examination showed high cellularity, the presence of pleomorphic nuclei, and areas of necrosis and diffuse neovascularization (Table S3 Supplementary Materials). The images reported in Figure 1 and Figure 2 depict the characteristics of glioblastoma IDH wild type. Figure 1A shows a highly cellular tumor in which microvascular proliferation is evident in the center of the microscopic field. The image in Figure 1B shows the immunohistochemical staining results for IDHR232H, a genetic marker commonly used to identify glioblastomas with IDH mutations. The neoplastic cells are negative for this marker, indicating that they do not carry this specific mutation. Figure 1C,D provide the control, representing a glioblastoma IDH mutant with the same staining as Figure 1A,B.

Figure 2A illustrates the immunohistochemical staining for EGFR. As it is visible from the orange staining, the neoplastic cells are positive.

The images in Figure 2B,C show the results of the immunohistochemical staining for Ki67 in two different samples: B shows a sample of our patient group, and many orange-stained cells are visible, indicating high proliferative activity. In Figure 2C, a control sample with few and scattered, orange-stained cells showed low proliferative activity.

### 3.2. Light Microscopy Analysis of Glioblastoma Tissue

**Figure 3:**
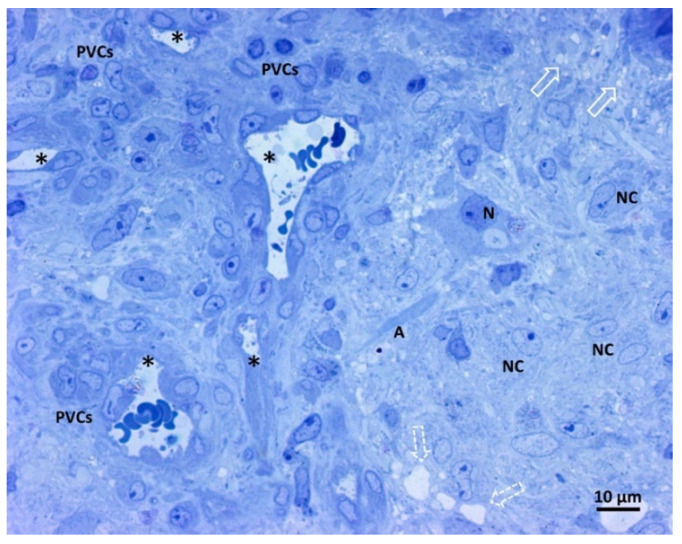
Light microscopy image of methylene blue stained semithin section. Magnification 400×, bar 10 microns. This picture shows a panoramic view of glioblastoma tissue. Neoplastic cells of glial origin (NC), vessels sections (*). A neuron (N) and its axon (A) are interspersed among fibrillar material and neoplastic cells. Small intercellular spaces (empty arrow), more extensive areas (open dotted arrow), and perivascular neoplastic cells (PVCs).

Figure 3 shows our samples’ comprehensive glioblastoma architecture overview. The numerous visible blood vessel sections show a high level of vascularization. In particular, two transverse and round-shaped sections, two “Y” shaped sections indicative of branching vessels, and one longitudinal section are present. The histological variability of glioblastoma is clear from the left and right parts of the image, with a range of neoplastic cells displayed. The right side of the image contains neoplastic cells with large oval nuclei, dispersed chromatin, one prominent nucleolus, and pale cytoplasm. On the left side of the image, perivascular neoplastic cells are visible, arranged around small blood vessels with darker cytoplasm, irregularly shaped nuclei, and less dispersed chromatin. A neuron and its axon interspersed among fibrillar material and neoplastic cells were also present, as well as small and large intercellular spaces.

**Figure 4:**
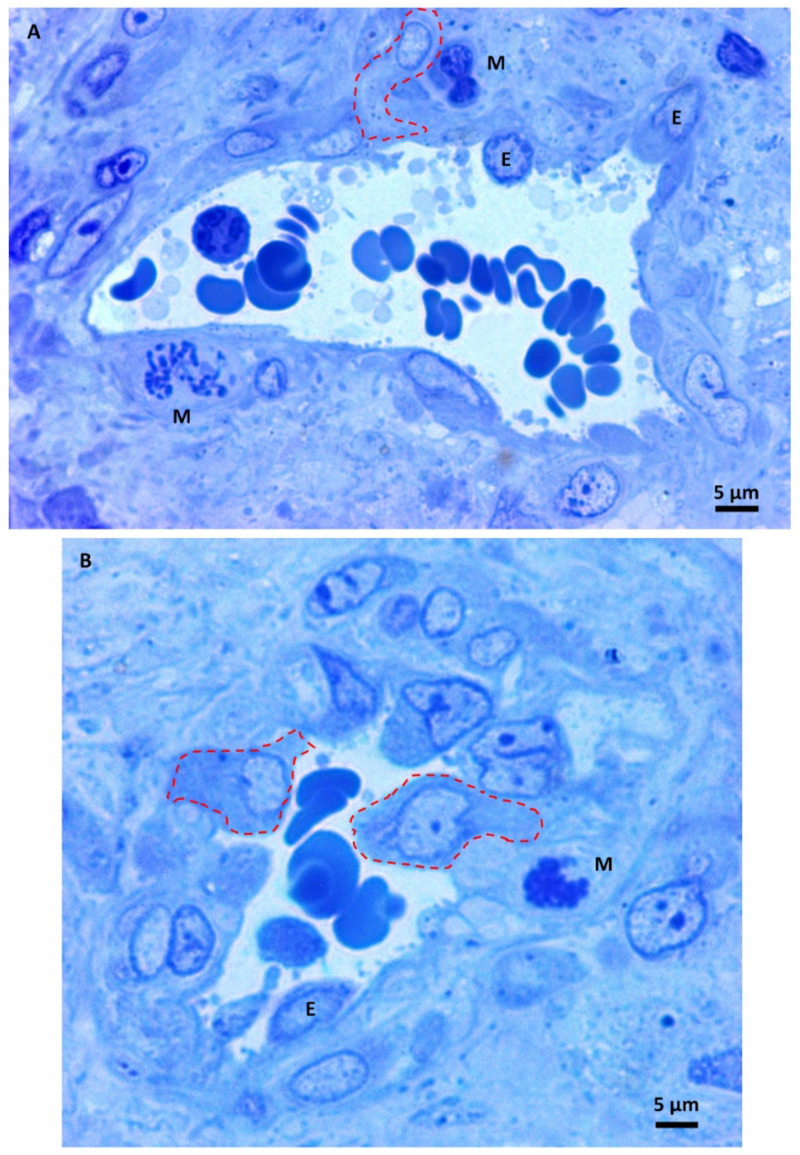
(**A**,**B**) Light microscopy images of methylene blue stained semithin section. Magnification 1000×, bar 5 microns. Hyperplastic endothelial cells (E). Mitotic figures (M) are sometimes very close to the endothelium—intravasating neoplastic cells (dotted lines).

Under higher magnification, the small glioblastoma vessels appeared irregular, and the triangular-shaped section in Figure 4A is a sign of vessel branching. They showed endothelial cell proliferation with many hyperplastic endothelial cells bulging into the lumen with oval or round shapes. The perivascular area contains mitotic figures (Figure 4A,B), sometimes close to the endothelium. Neoplastic cells moved towards the vessel and intravasation into its lumen (dotted lines). The above images visually represent the so-called perivascular niche, the microenvironment in which the molecular cross-talk between endothelial cells, astrocytes, and glioblastoma stem cells fuels the production of cells with invasive properties [38,39,40].

### 3.3. Transmission Electron Microscopy Analysis of Glioblastoma Tissue

#### 3.3.1. Small Vessels

**Figure 5:**
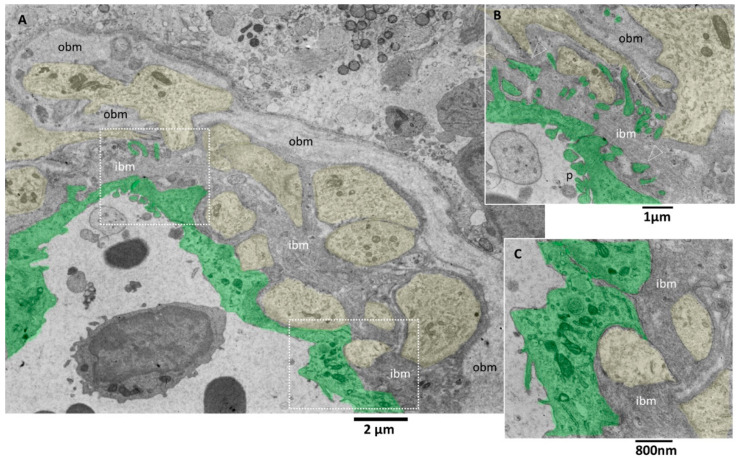
TEM, Glioblastoma small vessel wall. (**A**) Panoramic view of the vessel wall; note that a lymphocyte is visible in the lumen’s endothelial cells (green). The vessel basement membrane appears thickened, divided into two layers (inner basement membrane, ibm; outer basement membrane, obm), and penetrated by foot processes of astrocytes (yellow). (**B**) Endothelial cells (green) present cauliflower-like projections on their luminal surface (p), whereas the basal surface expresses long and deep finger-like projections (arrowheads) that penetrate the basement membrane to reach the astrocytes foot processes (yellow). (**C**) Astrocytes’ foot processes (yellow) appear separated by the endothelial cells (green) by an excellent layer of ibm fibers.

Transmission electron microscopy images allow more detailed visualization of the wall of glioblastoma small vessels (Figure 5A–C). They were lined by hyperplastic endothelial cells lying on a markedly thickened basement membrane. The luminal surface of many endothelial cells presents various bulbous cytoplasmic projections with wide stalks (cauliflower-like) and thin and long arms, which was visible projecting into the vessel lumen (Figure 5B). According to [41], the increased villous projections from the luminal surface of endothelial cells of glioblastoma vessels increase the surface area that actively resorbs the intravascular fluid.

The tight junctions between endothelial cells are well preserved. Interestingly, long projections develop from the abluminal aspect of endothelial cells, crossing the basement membrane’s total thickness. They head towards astrocytes’ footplates, contacting and wrapping around them. The basement membrane appeared formed by two layers, a more compact and darker one near the endothelium (inner basement membrane) and a looser and paler one distant from the endothelium (outer basement membrane). The basement membrane is discontinuous, and through its fenestrations, astrocytes’ foot plates arrive very near the basal aspect of the endothelial cells (Figure 5A–C). A lymphocyte is visible in the vessel lumen.

**Figure 6:**
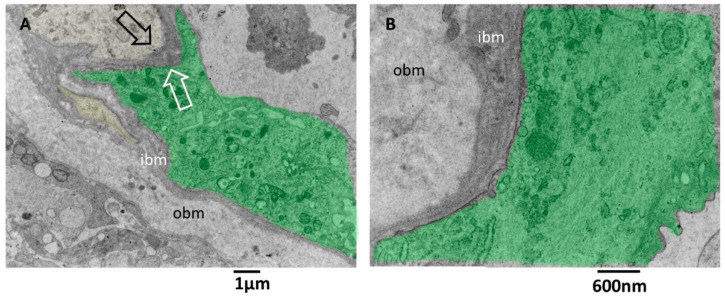
TEM, Glioblastoma small vessel wall, the basement membrane. (**A**) The endothelial cell (green) is separated by a skinny layer (black and white arrows) of ibm from the astrocyte foot process (yellow). (**B**) Endothelial cell (green) lies on a two-layered basement membrane. The outer layer (obm) filaments appear loosely arranged and longitudinally oriented. In the inner layer (ibm), filaments appear compact and circularly oriented.

Pictures captured at higher magnifications show the different layers of the basement membrane (Figure 6A,B). Astrocytes’ foot plates were separated by the basal aspect of the endothelial cells through a thin layer of ibm (Figure 6A). The ibm is formed by thicker filaments circularly arranged and surrounded by a dark amorphous matrix. The ibm consisted of thinner and loosely organized filaments, predominantly oriented longitudinal, following the vessel length (Figure 6B).

There is still a debate in the literature on the alteration of vascular basement membrane in glioblastoma; our images confirm the findings of [42] and add further ultrastructural details of the basement membrane in alteration in GMB. The vascular basement membrane appears structured in two layers, an innermost and an outermost, with different filament arrangements. From our images, the basement membrane fenestrations across which the astrocyte foot processes arrive very near the inferior aspect of endothelial cells; this proximity is needed to realize the cross-talk with endothelial cells. In addition, the close contact between endothelial cell processes and astrocytes is presented. These characteristics allow the passage of lymphocytes, as demonstrated by [42], and as is visible in our images.

#### 3.3.2. Neoplastic Cells

**Figure 7:**
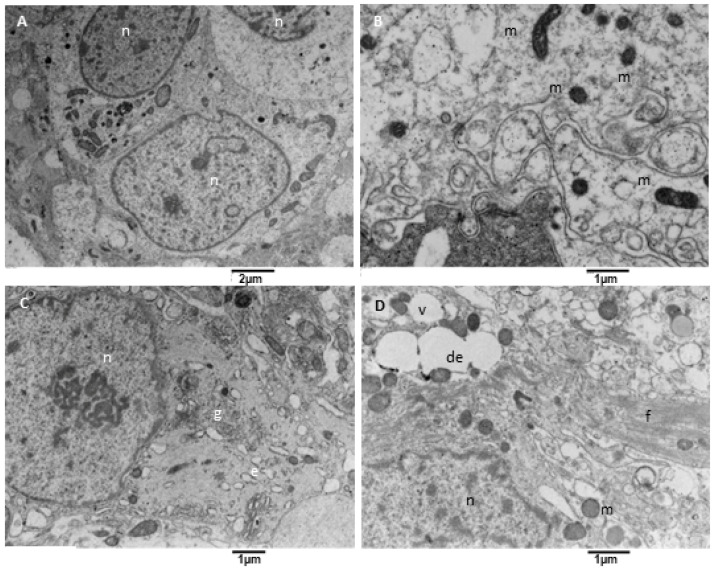
TEM, Neoplastic Cells. (**A**) The figure shows neoplastic cells (n) with a centrally located oval nucleus and cytoplasm rich in organelles, such as mitochondria. (**B**) Mitochondria (m) appear small and with a dense and dark matrix. (**C**) Some neoplastic cells show a well-developed Golgi apparatus (g) and moderately developed endoplasmic reticulum (e). (**D**) Some other cells contain glial filaments in the cytoplasm (f), mitochondria (m), vacuoles (v), and dilated endoplasmic reticulum (de).

Glioblastoma is characterized by significant variability in its neoplastic cell population; our samples mainly consist of round astroglia-derived cells with different and altered structural characteristics. We observed neoplastic cells with an oval-shaped nucleus occupying the cell center, with one or two evident nucleoli. The cytoplasm contains a variable amount of endoplasmic reticulum and mitochondria (Figure 7A). At higher magnification (Figure 7B) is apparent that mitochondria were small and had a dark matrix, and the endoplasmic reticulum is sometimes enlarged. Many cells present a large cell body with a well-formed Golgi apparatus, sometimes more than one (Figure 7C), and variable content of glial filaments (type of intermediate filaments, glial filaments have been morphologically identified on TEM images according to [43]). In particular, the organelles’ content decreases as the filaments amount increases. Glial filaments are interspersed in the neoplastic cell cytoplasm or can group in bundles (Figure 7D); vacuoles and enlarged endoplasmic reticulum were also present.

#### 3.3.3. Neoplastic Cells Mitophagy (NCsM)

**Figure 8:**
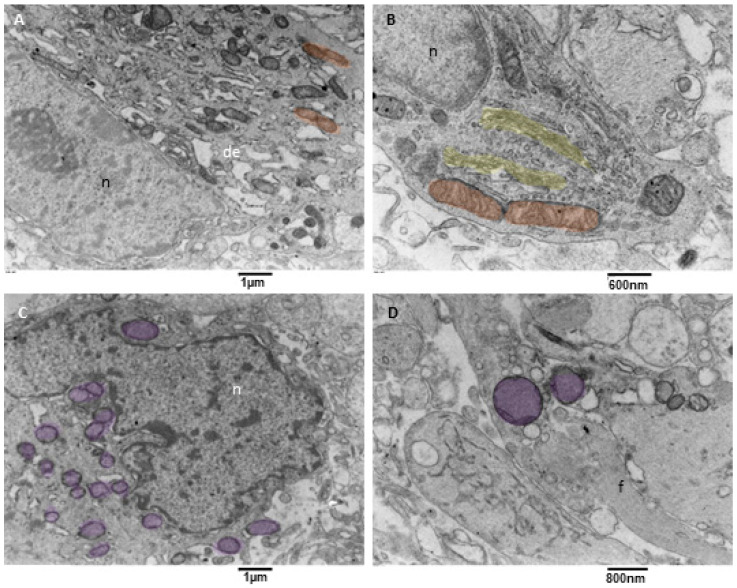
TEM, mitophagy in neoplastic cells. (**A**) A neoplastic cell is depicted. It is visible in the nucleus (n) and a prominent nucleolus. The cytoplasm contains dilated endoplasmic reticulum (de), and active mitochondrial fission (orange) can be observed. (**B**) This neoplastic cell shows a mitochondrion (orange) in the final stages of fission. A duplicated Golgi organ (yellow) is present. (**C**) A neoplastic cell with multiple mitochondria in advanced stages of mitophagy (violet) is represented. (**D**) At high magnification, two mitophagy figures (violet) are presented; note the cristae remnants inside the left mitophagy mitochondrion. Glial filaments (f) are also present in the cell cytoplasm.

Mitochondrial fission and mitophagy were observed in our samples, but with a variable degree since mitophagy was almost absent in one sample. This result is consistent with what happens in glioblastoma cells, in which an impairment of mitochondrial activity and autophagy suppression occurs; this drives stemness and invasion and indicates poor prognosis [44,45,46]. Our observation showed that neoplastic cells with active mitophagy had numerous cytoplasm organelles and sometimes glial filaments. Often, duplicated Golgi organs involved in active vesicle trafficking were present, the endoplasmic reticulum appeared to have a low/mild level of dilatation, mitochondria were actively dividing, and numerous mitophagy figures at different stages were also observed (Figure 8A–D).

#### 3.3.4. Microglia Cells

**Figure 9:**
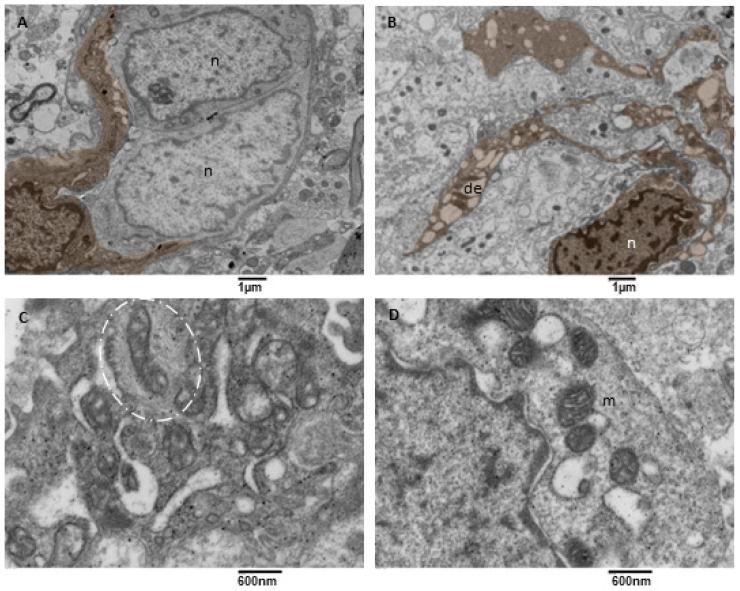
TEM, Microglia Cells. (**A**) A microglia cell (brown) surrounds two neoplastic cells (n) with two long processes. Bar 1 micron, (**B**) A microglia cell (brown) presenting multiple long processes in which a dilated endoplasmic reticulum (de) is visible. (**C**) Detail of a microglia cell cytoplasm showing mitochondria entrapped in a network of filaments (oval dotted line). (**D**) Detail of a microglia cell cytoplasm showing mitochondria (m) tethering with the dilated endoplasmic reticulum (de).

Microglia cells are considered the tissue-resident macrophages of the central nervous system; they help maintain brain homeostasis, acting as the first line of defence against pathogens or threats [47,48,49]. Microglia are motile and dynamic cells whose constant expansion and retraction of processes allow them to monitor the surrounding parenchyma. Microglia are essential in neuroinflammation; they are the principal source of inflammatory mediators in the nervous system (cytokines, ROS, and soluble lipids, as reported in [50]). We observed microglia cells characterized by a soma with an oval/elongated nucleus, a small ring of cytoplasm surrounding the heart, and multiple long and tortuous cellular processes (hyper-ramified/hypertrophic according to [51], Figure 9A,B). Their endoplasmic reticulum appeared moderately dilated (a sign of moderate stress), and often mitochondria in tethering were observed (Figure 9D). Their mitochondria showed mild signs of stress (cristae disorganization) and a dark matrix. The abnormal mitochondrial ultrastructure could underlie the shift in energy-generating pathways in glioblastoma for cell survival and progression. Mitochondria entrapped in a network of intermediate filaments were also observed (Figure 9C). A close examination evidenced that intermediate filaments in a part bind to the mitochondria, in part, are arranged concentrically in a confinement scenario, as described by [52]. Rare dark microglia cells were observed, according to [53,54].

#### 3.3.5. Microglia Cells Secreting sEVs (MsEVs)

**Figure 10:**
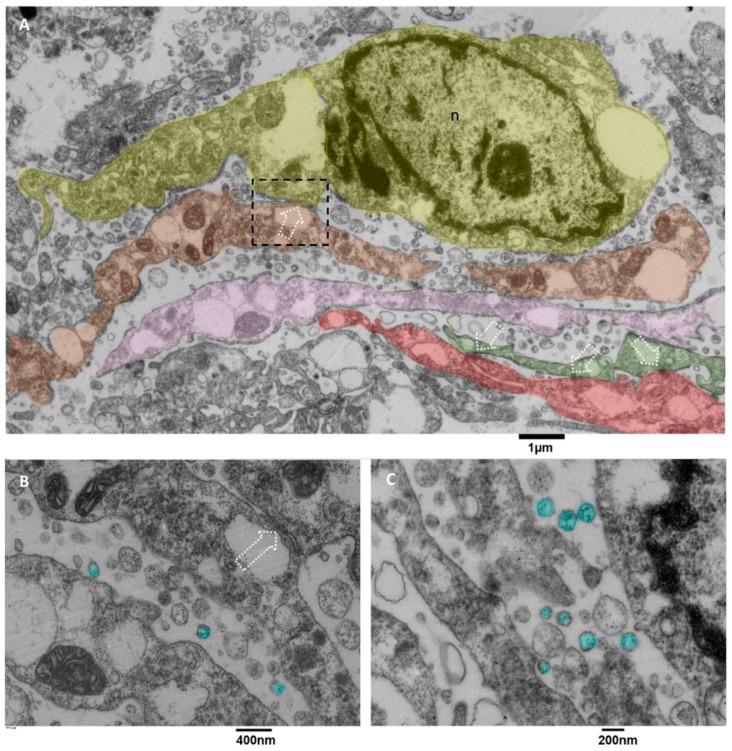
TEM, Microglia Cells. (**A**) panoramic view of at least five different microglia cells, represented in different colors (yellow, orange, pink, green and red), the dotted square representing a contact zone between two different microglia cells are enlarged in B, dotted arrows indicate the tight junctions between different cells, the endoplasmic reticulum appears dilated, sometimes severely. (**B**) The tight junction that connects two microglia cells is indicated with a dotted arrow; EVs are colored in light blue. Mitochondria show moderate signs of stress, with dilated cristae and a dark matrix; the endoplasmic reticulum is dilated. (**C**) the massive EVs secretion (light blue) is represented, and small vesicles are also visible in the cell’s cytoplasm.

In literature, microglia cells are involved in glioblastoma [18], and it is also reported that sometimes they arrange in clusters [55] whose formation is due to the activity of glioblastoma cells. Briefly, glioblastoma cells initially secrete low levels of the chemokine CCL2 to attract microglia cells chemotactically, and then also microglia cells start to release EVs containing CCL2 [56], attracting even more microglial cells into the tumor, promoting tumor progression and development, as stated in [55,57,58]. The value of our ultrastructural observations is that our images show ex vivo and in situ the EVs secretion by microglia cells. Usually, this activity is demonstrated in vitro by analyzing the culture medium with methods like Nanosight tracking analysis. Our images show the presence of clusters of ramified microglia cells connected by tight junctions (Figure 10A) and actively secreting massive amounts of EVs with diameters less than 200 nm. Those cells showed dilated endoplasmic reticulum and mitochondria with moderate stress (Figure 10B,C).

#### 3.3.6. Microglia Cells Storing Lipid Vesicles (MsLVs)

**Figure 11:**
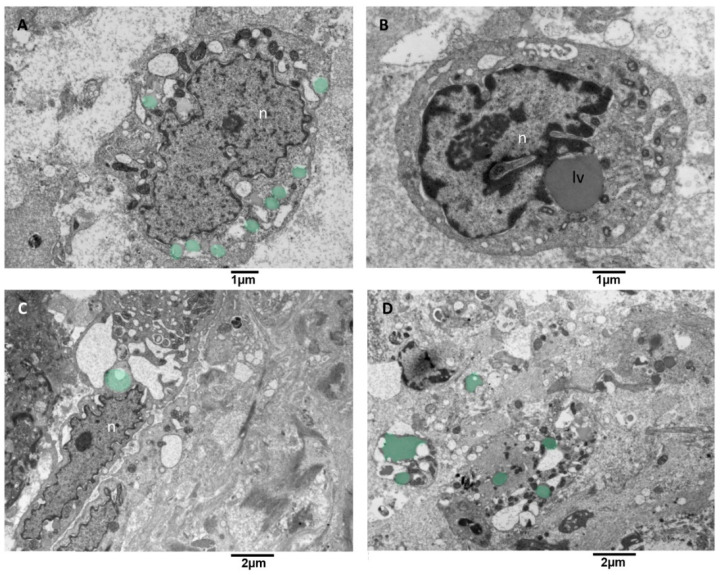
TEM, Microglia cells with lipid vesicles. (**A**) in this cell, multiple small lipid vesicles (green) surround the nucleus (n), and the endoplasmic reticulum appears enlarged. (**B**) this cell presents a single large lipid vesicle (lv) in tethering with the nucleus (n). (**C**) microglia cell with an elongated nucleus and severely dilated endoplasmic reticulum is shown; note the lipid vesicles in green near the nucleus (n). (**D**) This panoramic view shows the cytoplasm of different cells containing lipid vesicles (green).

Lipid droplet formation is undetectable in low-grade gliomas. The studies [59,60] reveal that their presence matches with progression to advanced-stage glioblastomas, which makes them a prospective biomarker and metabolic target in glioblastoma. We observed microglia cells storing lipid vesicles in the cytoplasm; sometimes, the lipid vesicle was single and large. More often, multiple lipid vesicles in a different emptying stage were present (Figure 11A). Lipid vesicles were in contact (tethering) with the nucleus but more often with the mitochondria (Figure 11B,C). The endoplasmic reticulum showed moderate dilatation; mitochondria appeared with mild signs of cristae disorganization and dark matrix (Figure 11D). This kind of microglia cells didn’t show EVs secretion activity.

#### 3.3.7. Statistical Analysis

**Figure 12:**
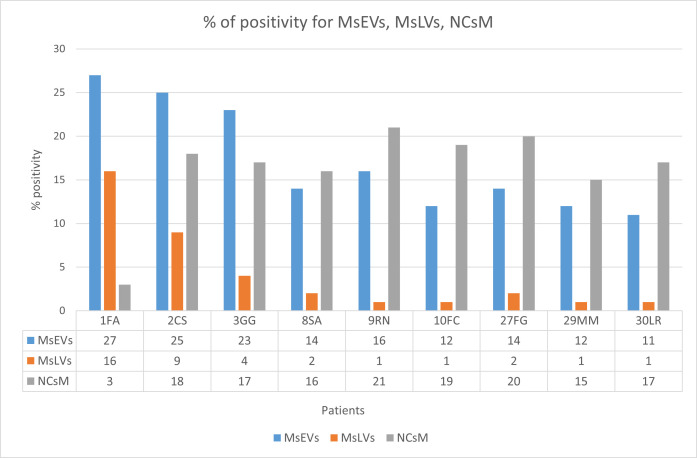
Percent of positivity for MsEVs (blue), MsLVs (orange), and NCsM (gray) in each patient. Note that high values of MsLVs are present in the same patient with high MsEVs. Values of NCsM appear instead almost constant among patients.

The percentage of positivity for microglia cells secreting sEVs (MsEVs), microglia cells storing lipid vesicles (MsLVs), and neoplastic cells with mitophagy (NCsM) were reported for each patient in Table S4 of the Supplementary Materials section and in Figure 12. Mean ± SD for MsEVs was 17.11 ± 6.17 with 95%CI = 12.36–21.85; Mean ± SD for MsLVs was 4.11 ± 5.15 with 95% CI = 0.14–8.07; Mean ± SD for neoplastic cells mitophagy was 16.22 ± 2.30 with 95% CI = 12.14–20.30.

**Figure 13:**
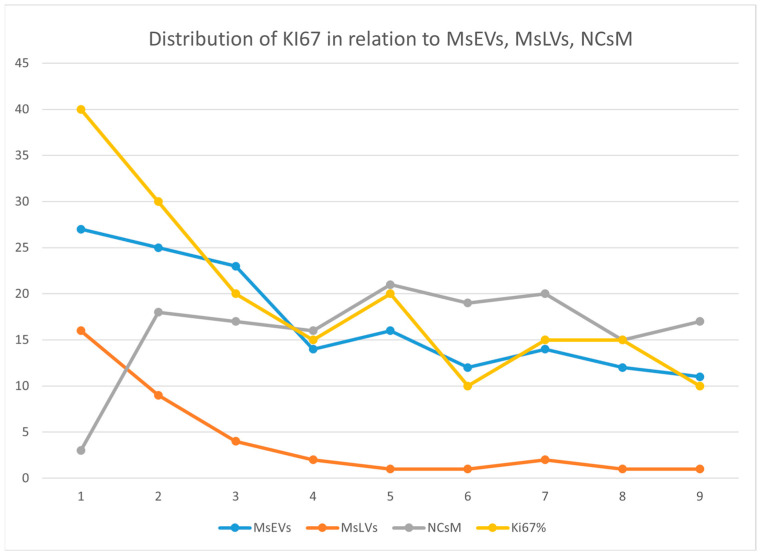
This figure illustrates the distribution of Ki-67 (yellow), MsEVs (blue line), MsLVs (orange line), and NCsM (gray line). High values of Ki-67 are related to high values for both MsEVs and MsLVs. Values of NCsM appear not related to Ki-67, having its value distribution line a different trend than that of Ki-67.

Being Ki-67, the only molecular parameter differing among our patients, we aimed to verify the existence of any correlation between this parameter and the ultrastructural ones. So, we plotted Ki-67 values (see Table S4 of Supplementary Materials section) together with the values of MsEVs (blue line), MsLVs (orange line), and NCsM (gray line). and the plot is shown in Figure 13.

**Figure 14:**
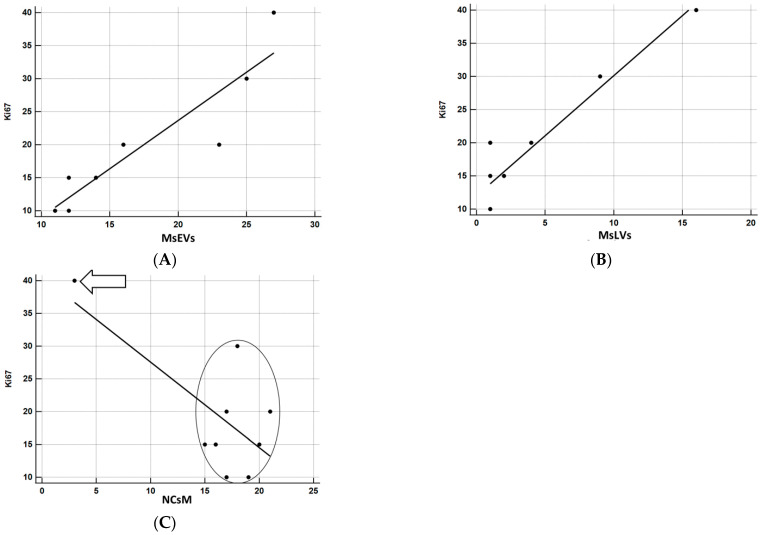
(**A**) Correlation analysis between Ki67 and MsEVs. r = 0.918, *p* < 0.0001; this result demonstrates the strong association between these two parameters, as it also appears clear by the regression line orientation the amount of MsEVs increases with increasing Ki-67. (**B**) Correlation analysis between Ki67 and MsLVs. r = 0.951, *p* < 0.0001; even in this case demonstrates the strong association between these two parameters, the regression line indicates that as the amount of MsLVs increase, the same is for Ki-67. (**C**) Correlation analysis between Ki67 and NCsM. r = −0.704, *p* < 0.05. Arrow: a single subject with very high scores in KI67 and very low scores in mitophagy. In the circle, all other subjects were comprised. A clear pattern of association between the two variables is not present in this case.

The relationship between KI-67 and the other parameters was investigated using correlation coefficients and graphically represented using the scatterplot. Being the sample particularly small, to have a more solid estimate of the correlation, bootstrap estimates were obtained using SPSS facilities, setting the number of replicated bootstrap samples to 10,000. Bootstrapping is particularly useful relative to smaller samples, especially in the presence of outliers [61]. The results of correlation analyses are precise (Figure 14A–C). MsEVs are highly correlated with Ki67, r = 0.918, *p* < 0.0001 (the 95% confidence interval limits for the bootstrap estimates are 0.776; 0.996). Figure 14A confirms the almost perfect linear relation between the two variables. MsLVs are also strongly correlated with Ki67, r = 0.951, *p* < 0.0001 (the 95% confidence interval limits for the bootstrap estimates are 0.290; 0.994). As shown in Figure 14B, the linearity of this relation is self-evident, albeit the presence of a subject whose score on Ki67 is somewhat higher concerning what expect from his very low score on MsLVs: this is probably the cause of a much wider confidence interval. Finally, although the negative correlation of NCsM is solid and significant, being r = −0.704, *p* < 0.05, the 95% confidence interval limits for the bootstrap estimates are −0.958; 0.468, being 0 a possible value with 95% of confidence, we cannot reject the null hypothesis that this correlation is 0. Scrutiny of the scatterplot (Figure 14C) shows that the negative correlation is almost uniquely due to a single subject with very high scores in Ki67 and very low scores on mitophagy: the other subjects are scattered without evidencing a clear pattern of association between the two variables.

## 4. Discussion

The classification of CNS tumors has traditionally relied on histological observations and tests performed on tissue samples. However, the most recent version of the WHO Classification of Tumors of the Central Nervous System introduced a new approach. It now includes a wide range of molecular biomarkers that have proven clinically helpful and are crucial in accurately classifying CNS neoplasms.

In this new nomenclature, the presence in glioblastoma of a mutation in the enzyme IDH is introduced, and the once-named glioblastoma grade IV is now defined as Glioblastoma IDH-wild type [62,63,64,65,66].

Additional genetic and molecular indicators have recently been discovered to connect with glioblastoma. These include ATRX mutation [67,68], TP53 expression [69,70,71], GFAP [72,73], and EGFR [74,75,76,77]. According to the Supplementary Data in Table S2, our samples exhibit the wild type for IDH and ATRX, but they are positive for TP53, GFAP, and EGFR. The only molecular aspect that varies among our samples is the KI-67 LI, which represents a measure of cell proliferation.

KI-67 is evaluated using the monoclonal antibody Ki-67 (that stains nuclei reacting with nuclear proteins expressed in the cell cycle’s GI, S, G2, and M phases), and the nuclear positivity found for Ki-67 is expressed in percentage, KI-67 labeling index (LI). Ki-67 expression ranges from 15% to 40% in most glioblastomas, and high Ki-67 expression is related to lesion volume, an increased risk of recurrence, and a poor prognosis. [78]. According to our previous work [79], there is a correlation between the percentage staining of Ki-67 and overall survival in patients with IDH-WT glioblastoma; specifically, the percentage of Ki-67 staining >20% predicts poorer progression-free survival. However, there is still a debate on this in the literature and existing papers that report different results [15].

Our work aimed to perform an ultrastructural analysis of glioblastoma specimens with the same histological and molecular profile but different Ki-67 to find possible morphological characteristics related to Ki-67, but not to express any relationship with the overall survival.

We summarize and discuss the results of our study in five points:

1. Visualization of the anatomical basis of astrocyte-endothelial cells’ crosstalk

During our observations, we identified micro vessels whose basement membrane (consisting of two layers) presented small openings called fenestrations. We observed that these fenestrations are crossed by astrocytes’ foot processes, facilitating their proximity to the neighboring endothelial cells. We also observed that the endothelial cells extended through these fenestrations long processes towards the astrocytes. This feature represents the anatomical basis of their molecular crosstalk that, as demonstrated in a 3D coculture spheroids, significantly enhances tumor cells’ stemness marker expression [80]. The signaling pathway between astrocytes and the endothelial cell barrier in mice in an orthotopic model of human glioblastoma [81] demonstrated the protection of cancer tissue from chemotherapeutic agents given by these cells. Moreover, altering vessels’ basement membranes provides pathways for tumor growth and invasion and enables the entrance of immune cells that activate the astrocytes and promote neuroinflammation [82]. The alteration in the basement membrane could be exploited to develop new therapies.

2. Ultrastructural in situ imaging of clusters of hyperactivated microglia cells (MsEVs)

Our study of ex vivo samples demonstrates in situ the presence of clusters of hyperactivated microglia cells, contacting each other by tight junctions and secreting massive amounts of EVs.

In the context of glioblastoma, EVs released by tumor cells have been under study for the last ten years; neoplastic cells release EVs that contain high levels of the protein EG-FRvIII, which promotes tumor growth and invasion [83], EVs released by glioblastoma cells can induce angiogenesis, the process by which new blood vessels are formed, which is essential for tumor growth and progression [84,85]. EVs have also been implicated in therapy resistance in glioblastoma; EVs released by glioblastoma cells can transfer resistance to the chemotherapy drug temozolomide to recipient cells) and can induce resistance to radiation therapy [86]. Instead, EVs secreted by microglia are only recently investigated. They have been shown to contain a variety of inflammatory mediators, including cytokines and chemokines, which can be transferred to neighboring cells and modulate their activity. Some studies report that EVs from microglia cells attenuate glioblastoma proliferation. Still, the study of [87] clarified that only the spinal cord-derived EVs microglia under LPS stimulation significantly attenuated glioma proliferation, whether the cortical microglia were accounted for exerted anti-inflammatory effect. Neurogenesis/tumorigenesis properties are consistent with the relationship we found in our study. Our previous findings [53,54] show that microglia-secreted EVs influence the glioma microglia phenotype and modify tumor growth via transporting miR-124 to astrocytes and tumor cells. This miRNA modifies tumor metabolism by acting on glutamate homeostasis. More studies are needed to fully understand the genesis, the release mechanisms, and the effects of EVs in glioblastoma to develop targeted therapies that can modulate their formation and release.

3. Ultrastructural in situ imaging of microglia cells storing lipid vesicles (MsLVs)

We observed microglia cells storing lipid vesicles, active organelles that provide metabolic fuel, inflammatory intermediates, and signaling mediators to cells. The value of our images consists in the representation of microglia cells storing lipid vesicles in situ, in their in vivo tumor microenvironment, in which microglia cells express feels conditions and express a behavior (thus have a morphology) that it is not possible to recreate in vitro. Lipid vesicle biogenesis increases in neoplastic and inflammatory conditions. In literature is reported that microglia cells can accumulate lipid droplets in their cytoplasm, assuming a distinct phenotype called lipid droplet accumulating microglia [87]. The literature [88] reports evidence of a defective phagocytic phenotype in microglia cells storing lipid vesicles, in that enhanced phagocytic up-take of lipid exacerbates the lipid droplet accumulation burden and promotes chronic and self-sustained microglial activation [89]. In turn, sustained inflammation further pushes microglia into a hyperactivity state, creating feedback that exacerbates neuroinflammation and damages the integrity of the blood-brain barrier [90]. As our images demonstrate, the above-described overall scenario perfectly fits with our results. It reinforces our hypothesis of a relationship between the presence of microglia cells storing lipid vesicles and high levels of Ki-67LI.

4. Ultrastructural in situ imaging of neoplastic cells mitophagy (NCsM)

Our pictures evidenced the presence of mitophagy in neoplastic cells. In literature, this process is frequently triggered by oxidative stress or an increased need for energy, as it occurs during cancer growth and invasion [91]. Some research [92,93] investigated the involvement of mitophagy in gliomas. Mitophagy inhibition was shown to partially reverse cannabidiol-induced glioma cell death, indicating a favorable role for mitophagy in anti-tumor therapy [94]. Two investigations suggest that inducing mitophagy via FOXO3a protects gliomas against temozolomide-induced cytotoxicity, demonstrating that mitophagy has a double-edged sword effect on gliomas [95,96,97]. Thus, whether mitophagy is related to glioblastoma prognosis and the likely involvement of mitophagy-related genes need to be determined.

5. Statistical analysis of ultrastructural data

The statistical analysis of our semiquantitative data of the morphological biomarker (MsEVs and MsLVs, and NCsM) expression and their relationship with Ki67-LI was performed by bootstrapping, a procedure particularly useful to manage small samples [57] as it was in our case. The results indicate that the MsEVs and MsLVs strongly correlate with the Ki-67 LI value. We can thus assume they are good candidates to be considered morphological biomarkers correlating to Ki-67 LI (however, we are collecting specimens to increase the sample size).

The role of NCsM instead must be further evaluated, being absent a clear pattern of association. We underline the fact that observations on a larger sample size are needed, but based on the analysis we conducted, we can reasonably candidate MsEVs and MsLVs as morphological biomarkers related to the Ki67 index in adult glioblastoma IDH1.

The presence of such a correlation may be used to improve the diagnostic procedure by combining ultrastructural features with molecular information to provide a single, “integrated” diagnosis (in view of personalized medicine). It may be helpful to assess the aggressiveness and prognosis of the tumor (as a guide to treatment decisions and patient management). One further use of such biomarkers is that of potential therapeutic targets (revealing specific cellular structures and molecular pathways that are unique to the single individual tumor). Finally, their utilization may help develop personalized treatment plans; studying the ultrastructural characteristics of the tumor tissue from every single patient will allow the development of tailored therapies for the specific characteristics of the single and specific patient’s tumor.

## 5. Conclusions

Our study of adult IDH Wild-Type Glioblastoma explored its ultrastructural characteristics and their correlation with the Ki-67 labeling index (LI). The results of our research show that integrating ultrastructural features with molecular information allows us to identify biomarkers that may improve diagnostic accuracy, guide treatment decisions, identify therapeutic targets, and facilitate personalized treatment plans tailored to individual patients. Future investigations with larger sample sizes are necessary to validate these findings and harness the potential of ultrastructural analysis in glioblastoma management.

## Data Availability

Data are stored in The Electron microscopy unit of the Sapienza SAIMLAL department and are available upon written request.

## Supplementary Materials

The following supporting information can be downloaded at: https://www.mdpi.com/article/10.3390/biomedicines11071968/s1. Table S1. Patients’ data and diagnosis. Table S2. Molecular data. Table S3. Histological data. Table S4. Morphological data from picture analysis and relation with Ki-67 index.
