# Supplementary material for: Adult IDH Wild-Type Glioblastoma Ultrastructural Investigation Suggests a Possible Correlation between Morphological Biomarkers and Ki-67 Index
Source: Biomedicines. 2023 Jul 12;11(7):1968. doi: 10.3390/biomedicines11071968 (PMC10377045; doi:10.3390/biomedicines11071968)
Supplement: Supplementary file 1 [file biomedicines-11-01968-s001.zip › biomedicines-2431841-supplementary.pdf]

Supplementary Materials:

Table S1. Patients' data and diagnosis.

Case	Age	SEX	Diagnosis
1FA	55	M	Glioblastoma Grade IV CNS-WHO2021, IDH wild type
2CS	46	F	Glioblastoma Grade IV CNS-WHO2021, IDH wild type
3GG	65	M	Glioblastoma Grade IV CNS-WHO2021, IDH wild type
8SA	61	F	Glioblastoma Grade IV CNS-WHO2021, IDH wild type
9RN	49	F	Glioblastoma Grade IV CNS-WHO2021, IDH wild type
10FC	57	F	Glioblastoma Grade IV CNS-WHO2021, IDH wild type
27FG	61	F	Glioblastoma Grade IV CNS-WHO2021, IDH wild type
29MM	63	M	Glioblastoma Grade IV CNS-WHO2021, IDH wild type
30LR	49	M	Glioblastoma Grade IV CNS-WHO2021, IDH wild type

Table S2. Molecular data.

Case	IDH1 mutation	ATRX Mutation	TP53 expression	Ki67 %	GFAP	EGFR overexpression
1FA	No	No	Yes	40%	Positive	Yes
2CS	No	No	Yes	30%	Positive	Yes
3GG	No	No	Yes	20%	Positive	Yes
8SA	No	No	Yes	15%	Positive	Yes
9RN	No	No	Yes	20%	Positive	Yes
10FC	No	No	Yes	10%	Positive	Yes
27FG	No	No	Yes	15%	Positive	Yes
29MM	No	No	Yes	15%	Positive	Yes
30LR	No	No	Yes	40%	Positive	Yes

Table S3. Histological data.

Case	HPF Mitosis x/10	Cellularity	Nuclei	Neo-vascularization	Necrosis
1FA	8/10	Higher	Pleomorphic	Yes	Yes
2CS	7/10	Higher	Pleomorphic	Yes	Yes
3GG	6/10	Higher	Pleomorphic	Yes	Yes
8SA	4/10	Higher	Pleomorphic	Yes	Yes
9RN	8/10	Higher	Pleomorphic	Yes	Yes
10FC	6/10	Higher	Pleomorphic	Yes	No
27FG	6/10	Higher	Pleomorphic	Yes	Yes
29MM	3/10	Lower	Pleomorphic	Yes	Yes
30LR	3/10	Lower	Pleomorphic	Yes	Yes

Table S4. Morphological data from picture analysis and relation with Ki67 index.

Case	Ki67%	% of MsEVs	% of NCsM	% of MsLVs
1FA	40	27	3	16
2CS	30	25	18	9
3GG	20	23	17	4
8SA	15	14	16	2
9RN	20	16	21	1
10FC	10	12	19	1
27FG	15	14	20	2
29MM	15	12	15	1
30LR	40	11	17	1
